# *Euphilomedes biacutidens* (Ostracoda, Myodocopida, Philomedidae), a new species from China Sea

**DOI:** 10.7717/peerj.3488

**Published:** 2017-06-22

**Authors:** Peng Xiang, Youyin Ye, Xiaoyin Chen, Ruixiang Chen, Mao Lin

**Affiliations:** 1 Laboratory of Marine Biology and Ecology, Third Institute of Oceanography, SOA, Xiamen, China; 2 School of Life Science and Technology, Tongji University, Shanghai, China; 3 Collaborative Innovation Center of Deep Sea Biology, Hangzhou, China

**Keywords:** Taxonomy, Ostracoda, *Euphilomedes biacutidens* sp. nov., Taiwan Strait, South China Sea

## Abstract

Ostracods are one of the major groups of marine benthos, inhabiting virtually all oceanic environments worldwide, and a total of 31 species have been recorded in genus *Euphilomedes* Kornicker, 1967. In the present study, we describe a new species *Euphilomedes biacutidens* collected from the Taiwan Strait and South China Sea.* E. biacutidens* sp. nov. differs from the related species of the genus* Euphilomedes* in having a unique combination of the characteristics of spines on carapace, the filaments on sensory seta, the arrangement of setae on tip of the first antenna, the numbers of setae on appendages, the claws on fifth limb, the teeth on the comb of the seventh limb and furcal claws. It is particularly obvious that it has a bifurcated and pointed ventral corner of the rostrum, two spines on the posterior margin of right valve, a row of teeth along the inner margin of article 3 of the endopod of the second antenna, and some long claws instead of setae on the fifth limb.

## Introduction

Ostracoda is a class of the phylum Arthropoda (Martin & Davis, 2001). The ostracods are small bivalved aquatic crustaceans and can be benthos or plankton. Ostracods are one of the major groups of marine meiobenthos and also macrobenthos which inhabit virtually all oceanic environments worldwide with various feeding habits and high taxonomic diversity (Karanovic, 2010). Studies on ostracods from China began in the 1950’s on fossil species (Chang, 1955). About thirty years later, we initiated investigations of the taxonomy and ecology of living marine ostracods in China (Chen, 1982; Chen, 1984). So far, 237 species of recent marine ostracods have been recorded from the China Sea (Chen, 2012; Chen et al., 2015a; Chen et al., 2015b; Xiang et al., 2017).

The genus *Euphilomedes* Poulsen, 1962, belonging to the subfamily Philomedinae Müller, 1908, is the second largest subfamily within family Philomedidae Müller, 1906, which contains 31 species (Brandão et al., 2017). Until now, ten species have been recorded from Chinese waters (Tseng, 1970; Chen & Lin, 1995; Chen & Lin, 1997; Liu, 2008; Chen, 2012; Chen et al., 2015a; Chen et al., 2015b; Xiang et al., 2017): *E. corrugata* (Brady, 1897), *E. interpuncta* (Baird, 1850), *E. japonicus* (Müller, 1890), *E. longiseta* (Juday, 1907), *E. liuruiyii* Xiang et al., 2017, *E. multiangular* Chen et al., 2015a, *E. nodosa* Poulsen, 1962, *E. pentacanthos* Xiang et al., 2017, *E. sordida* (Müller, 1890), and *E. spinulosa* Chen et al., 2015b.

**Figure 1:**
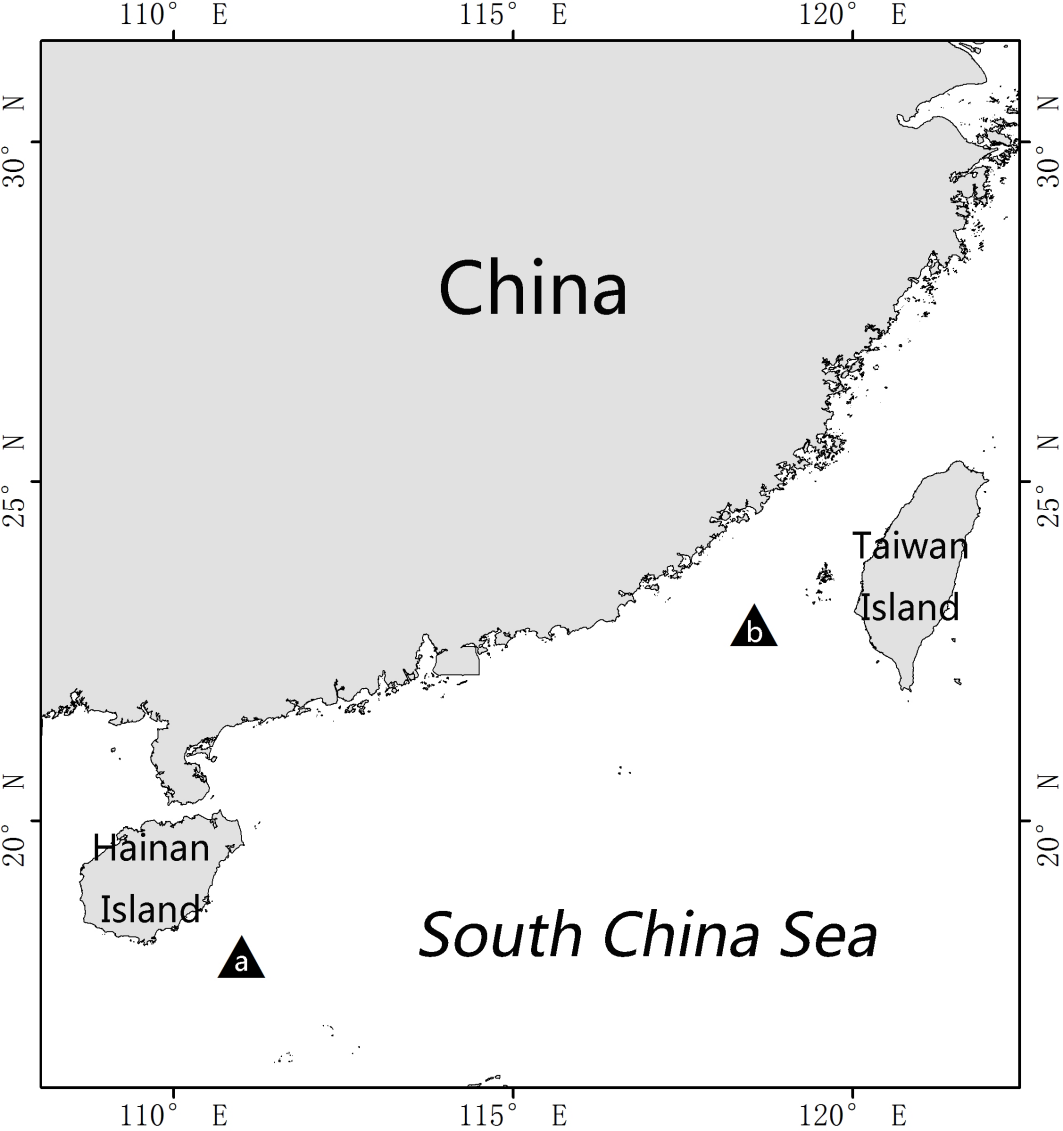
Sampling localities of *Euphilomedes biacutidens* sp. nov. (A) 18°N, 111°E, 1984 (B) 22°53′N, 118°33′E, 1994.

In the present study, we describe a further new species of *Euphilomedes* from the Taiwan Strait and the South China Sea (Fig. 1).

## Materials and Methods

Samples were obtained from two cruises of the South China Sea and Taiwan Strait in 1984–1985 and 1994–1995, respectively (Fig. 1). There are no specific permissions required for the sampling activities in the research areas.

All samples were collected using a sampling net with mouth diameter of 80 cm and a mesh aperture of 0.505 mm by vertical dragging from 200 m (or bottom) to surface water. Samples were fixed with 5% buffered formaldehyde for preservation.

Specimens were dissected under a zoom-stereomicroscope (Zeiss Discovery V2.0) and mounted in permanent slides with CMC-9AF mounting medium (Masters Company Inc., Wood Dale, IL, USA). Observations and photomicrographs were obtained with a transmitted-light binocular microscope combined with a differential interference contrast system and AxioVision Image-Pro software (Axio Imager Z2; Carl Zeiss Inc., Oberkochen, Germany). Line drawings were made from photomicrographs and observations of preserved specimens and dissected appendages in slides by Adobe Photoshop CS6 software (Adobe Inc., San Jose, CA, USA).

The type specimens were deposited in the Marine Biological Sample Museum of the Chinese Offshore Investigation and Assessment, the Third Institute of Oceanography, State Oceanic Administration, China (Xiamen, China), under the collection numbers TIO-OMPEu 326–TIO-OMPEu 329 for the new species.

### Nomenclatural acts

The electronic version of this article in Portable Document Format (PDF) will represent a published work according to the International Commission on Zoological Nomenclature (ICZN), and hence the new names contained in the electronic version are effectively published under that Code from the electronic edition alone. This published work and the nomenclatural acts it contains have been registered in ZooBank, the online registration system for the ICZN. The ZooBank LSIDs (Life Science Identifiers) can be resolved and the associated information viewed through any standard web browser by appending the LSID to the prefix http://zoobank.org/. The LSID for this publication is: urn:lsid:zoobank.org:pub:557FB253-93C6-473E-9E07-227D9D9C1A60. The online version of this work is archived and available from the following digital repositories: PeerJ, PubMed Central and CLOCKSS.

## Results

### Systematic account

**Table:** 

Order Myodocopida Sars, 1866
Family Philomedidae Müller, 1906
Genus *Euphilomedes* Poulsen, 1962

***Euphilomedes biacutidens*** **Xiang, Ye & Chen sp. nov.**

urn:lsid:zoobank.org:act:F773B126-7B58-45EA-8F28-8FCEB23B5868

Figs. 2–5.

**Figure 2:**
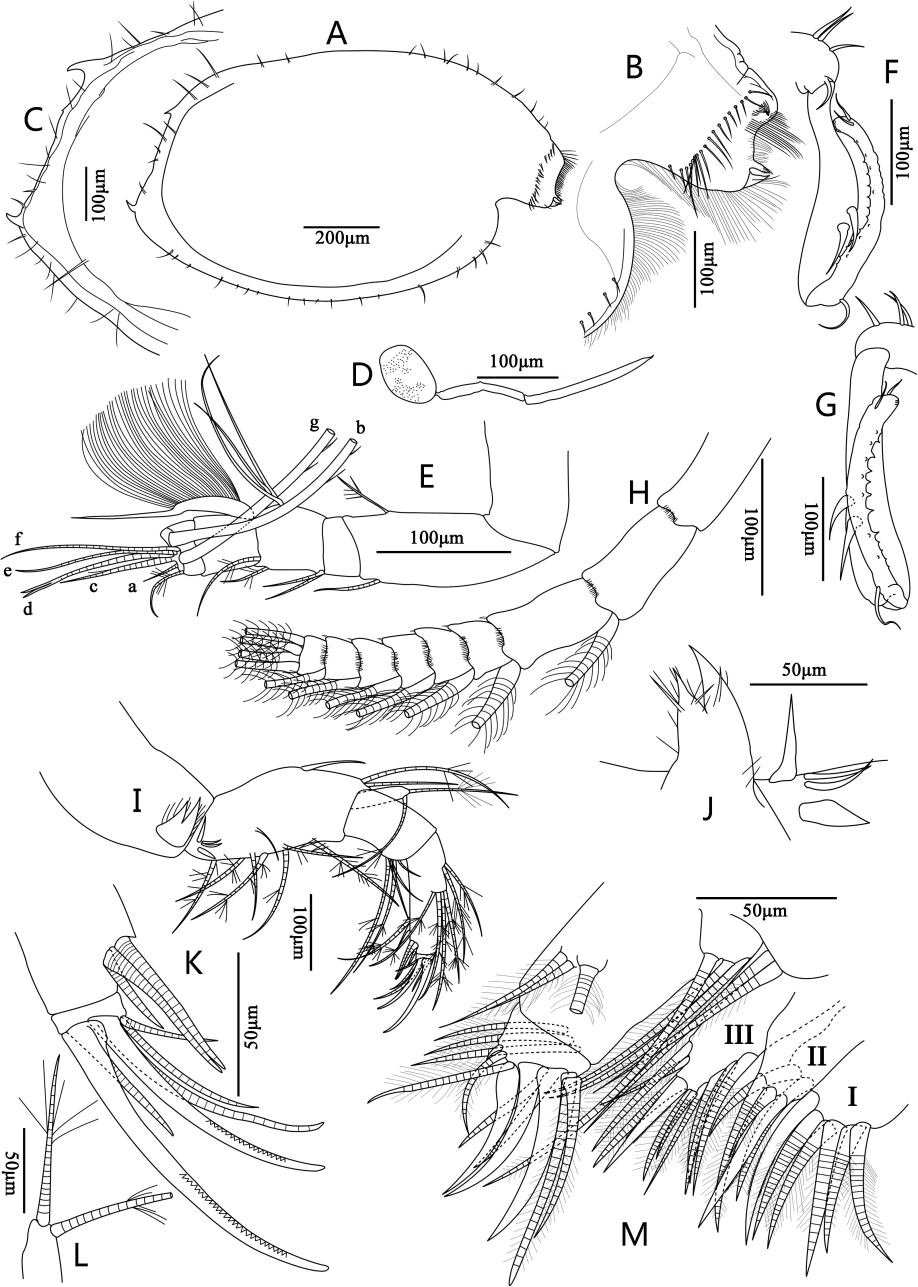
Line drawings of *Euphilomedes biacutidens* sp. nov., ♂. (A) Right valve, lateral view. (B) Rostrum, lateral view. (C) Posterior margin of right valve, lateral view. (D) Frontal organ, lateral view. (E) First antenna, lateral view. (F) Endopod of right second antenna, lateral view. (G) Endopod of left second antenna, lateral view. (H) Exopod of the second antenna, lateral view. (I) Mandible, lateral view. (J) Coxale endite, lateral view. (K) Tip of mandible, lateral view. (L) Exopod of mandible, lateral view. (M) Maxilla, lateral view.

**Figure 3:**
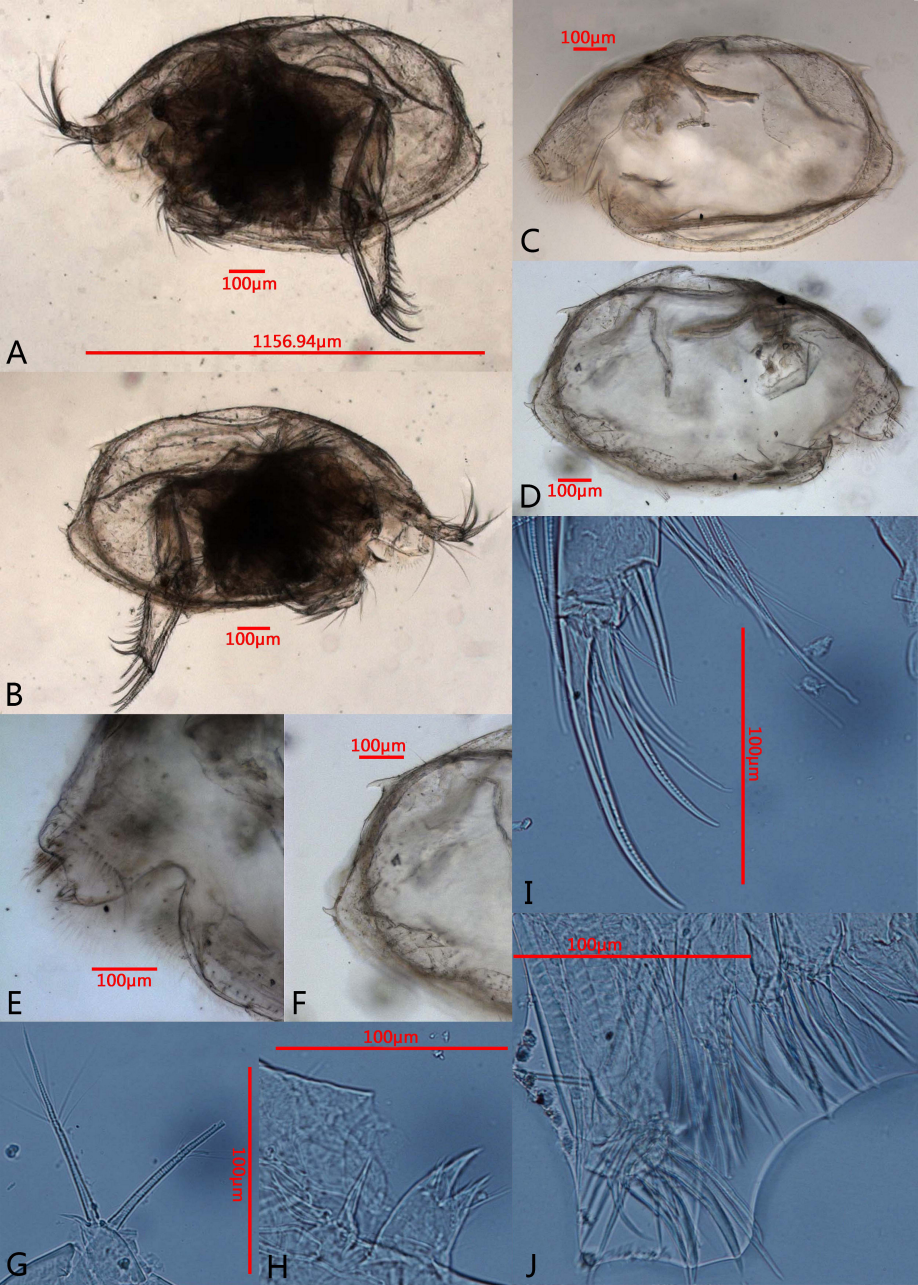
Photomicrographs of *Euphilomedes biacutidens* sp. nov., ♂. (A) Left valve, lateral view. (B) Right valve, lateral view. (C) Left valve, lateral view. (D) Right valve, lateral view. (E) Rostrum, later view. (F) Posterior margin of right valve, lateral view. (G) Exopod of mandible, lateral view. (H) Coxale endite, lateral view. (I) Tip of mandible, lateral view. (J) Maxilla, lateral view.

**Figure 4:**
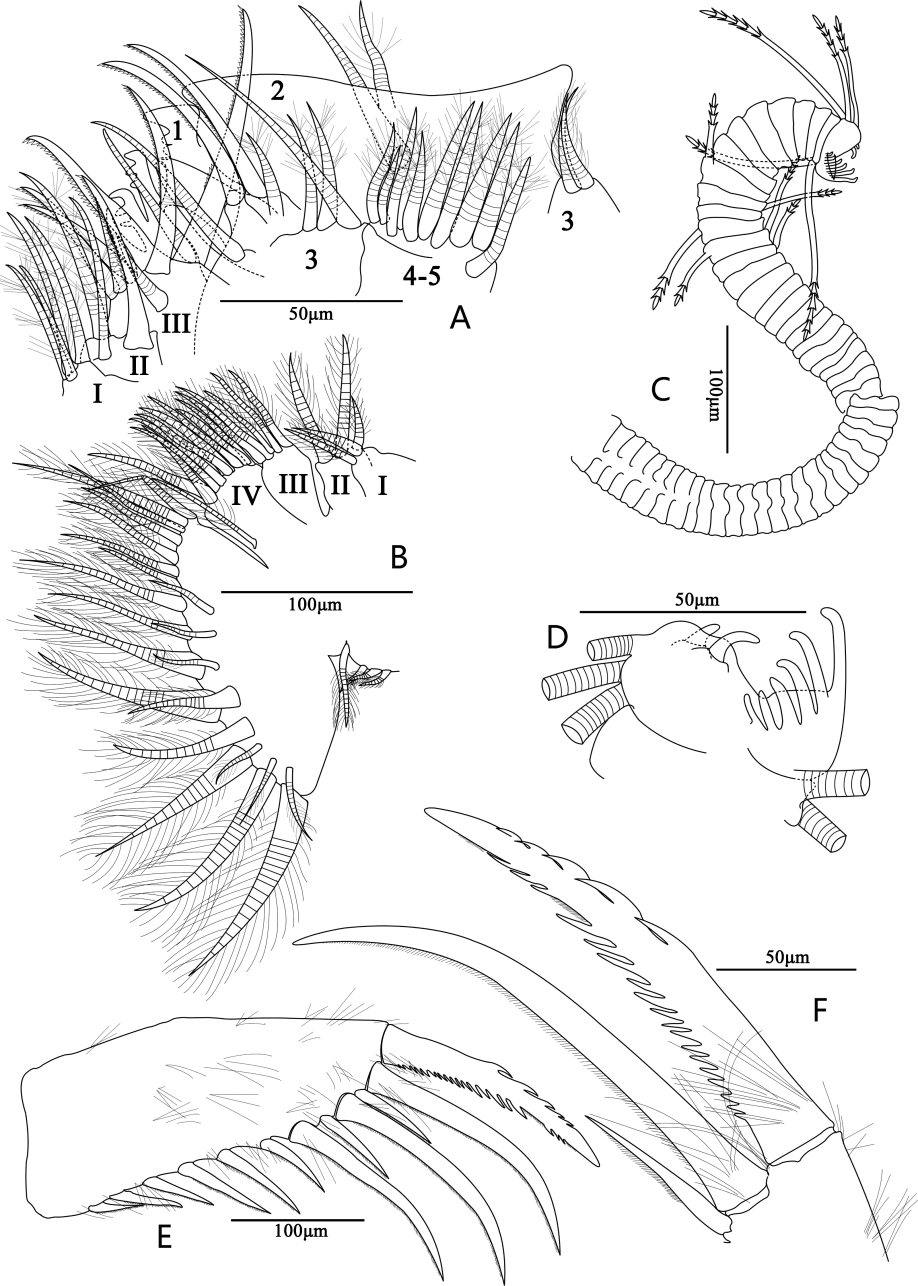
Line drawings of *Euphilomedes biacutidens* sp. nov., ♂. (A) Fifth limb, lateral view. (B) Sixth limb, lateral view. (C) Sixth limb, lateral view. (D) Comb of seventh limb, lateral view. (E) Furca, lateral view. (F) Detail of furca, lateral view.

**Figure 5:**
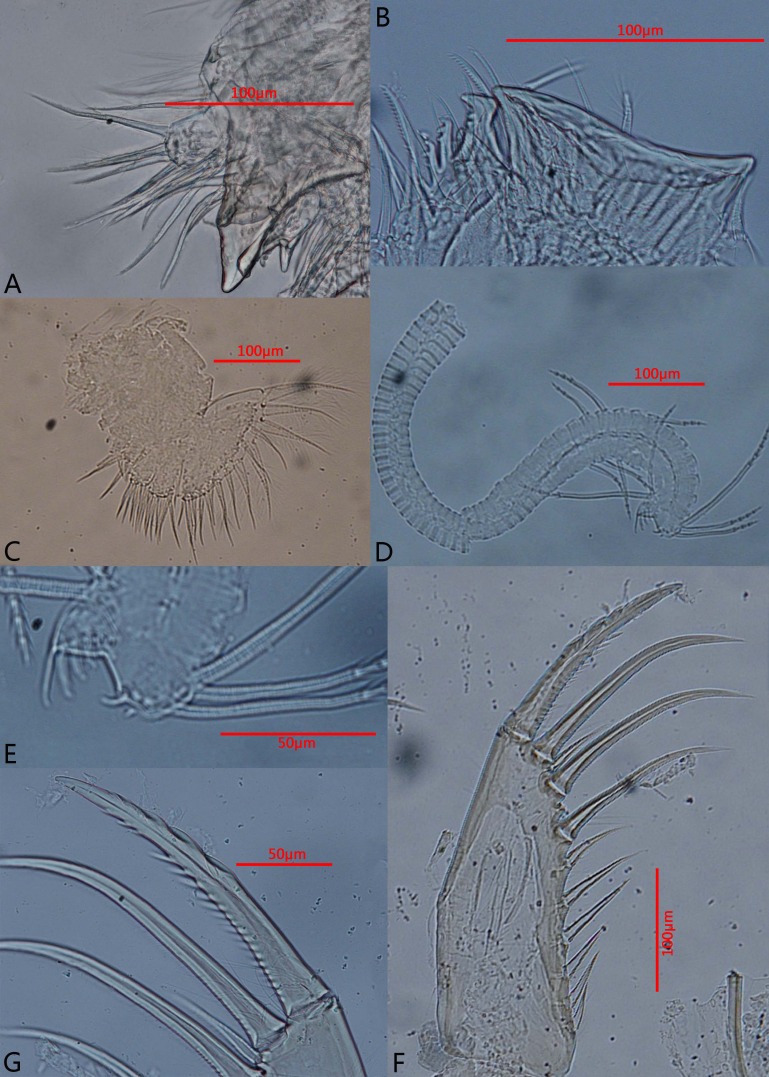
Photomicrographs of *Euphilomedes biacutidens* sp. nov., ♂. (A) Fifth limb, side view. (B) Fifth limb, lateral view. (C) Sixth limb, lateral view. (D) Seventh limb, lateral view. (E) Comb of seventh limb, lateral view. (F) Furca, lateral view. (G) Detail of furca, lateral view.

**Etymology.** *biacutidens*, derived from Latin expression of two spines, indicates that this species has one dorsal spine and one ventral spine on posterior margin of right valve.

**Holotype.** No. TIO-OMPEu 326, adult male, length 1.16 mm, height 0.69 mm, dissected on slides, carapace in alcohol. Type locality: southeastern coastal waters of Hainan Island (18°N, 111°E), depth 102 m, 29 July 1984.

**Paratypes.** No. TIO-OMPEu 327, adult male, length 1.10 mm, height 0.66 mm, deposited in 5% buffered formaldehyde, collected in the same way as the holotype; No. TIO-OMPEu 328, adult male, length 1.19 mm, height 0.71 mm, dissected on slides, collected from the southern Taiwan Strait (22°53′N, 118°33′E), depth 42 m, 29 August, 1994; No. TIO-OMPEu 329, adult male, length 1.14 mm, height 0.69 mm, deposited in 5% buffered formaldehyde, collected in the same way as TIO-OMPEu 328.

**Distribution.** South China Sea and Taiwan Strait (Fig. 1).

**Diagnosis.** Height about 60% of length. Carapace oval, external surface with tiny circular pits and small setae (Figs. 2A–2C, 3A–3F). Rostrum projecting and broad, with rounded dorsal corner, bifurcated and pointed ventral corner, marginal cilia and one row of anterior setae; incisure deep with cilia (Figs. 2B, 3E). Postero-ventral corner projecting, posterior margin of right vale with dorsal and ventral spines (Figs. 2A, 2C, 3A–3D, 3F). Sensory seta of the first antenna with about thirty-two long filaments (Fig. 2E). Endopod of the second antenna with twelve ventral sawteeth on article 3 (Figs. 2F–2G). Mandibular coxale endite spinose with bifurcated tip (Figs. 2I–2J, 3H); mandible with two claws and four setae on tip (Figs. 2K, 3I). Seventh limb with eleven cleaning setae (Figs. 4C, 5D); comb with six teeth, side opposite comb with two small pegs (Figs. 4D, 5E). Furcal lamella hirsute; claws 1, 2, 4 and 6 primary, claws 3, 5, 7–12 secondary (Figs. 4E, 5F); main claw 1 with prominent dorsal and ventral sawteeth (Figs. 4F, 5G).

**Description.**

**Carapace**: Valves oval in lateral view, thin and transparent, with tiny circular pits and small setae over surface (Figs. 2A, 3A–3D). Rostrum broad with rounded dorsal corner and pointed bifurcated ventral corner, incisure deep, rostrum with dense marginal cilia and one row of anterior setae, incisure with numerous long cilia (Figs. 2B, 3E). Dorsal to posterior margin evenly rounded, antero-ventral margin distinctly rounded, ventral margin slightly rounded, postero-ventral corner projecting backward. Right valve with one spine on postero-dorsal corner and one spine on postero-ventral corner; left valve without spines (Figs. 2C, 3F). Carapace length 1.10–1.19 mm, height 0.66–0.71 mm, height about 60% of length, greatest height near mid-length.

**Frontal organ**: Frontal organ extremely long and thin with two articles, article 2 longer with a sharp tip (Fig. 2D).

**First antenna**: First antenna uniramous with eight articles (Fig. 2E). Articles 1 and 2 long. Article 2 with one disto-dorsal and one disto-ventral plumose setae. Article 3 short with two spinose setae on disto-dorsal margin. Article 4 with one long and one short plumose setae on disto-dorsal margin, three long setae on mid-ventral margin, and one grand sensory seta with about thirty-two very long soft ventral filaments on disto-ventral margin. Article 5 bare. Article 6 very short with one short and bent plumose seta on disto-dorsal margin. Article 7 and 8 fused, very small with seven setae on tip: a-seta very short, spinose b- and g-setae very grand and long, c-seta with one mid filament, d-seta long with bifurcated tip, e- and f-setae long and bare.

**Second antenna**: Second antenna biramous. Endopod with three articles (Figs. 2F–2G). Article 1 short with three short ventral dorsal setae and one disto-ventral seta; article 2 long and slightly bent with corpulent ventral part, and two grand setae on ventro-distal margin; article 3 thin and bent, approximately equilong to article 2, with one bent proximo-dorsal seta, two small distal setae, twelve small ventral acute teeth, rugged dorsal margin, uneven inner side, and ten small tines on tip. Exopod with nine articles (Fig. 2H). Articles 1–8 with a line of fine spines on medio-distal margin; articles 2–8 with one disto-vental long plumose swimming seta, respectively; articles 3–8 with one spine on disto-dorsal edge; article 1 very long, article 2–9 more and more shorter; article 9 very short with four long plumose setae on tip.

**Mandible**: Limb biramous (Fig. 2I). Coxale grand, endite with bifurcated tip and cluster of spines (Figs. 2J, 3H). Basale grand, dorsal margin slightly humped with one mid-dorsal seta and two distal plumose setae; ventral margin with a group of proximal short setae, seven plumose setae and one short medio-ventral seta. Exopod tiny with two equilong plumose setae on tip (Figs. 2L, 3G). Endopod with three articles. Article 1 with a group of five setae on disto-ventral margin (two long plumose and three short). Article 2 longer than one; dorsal margin with a group of two long proximo-dorsal setae (one bare and one plumose), a group of four mid-dorsal long setae; ventral margin with a group of two mid-ventral setae (one short and one long plumose), a group of three bare disto-ventral setae (inner one short, outer two equilong). Terminal article very small with two claws and four setae on tip (Figs. 2K, 3I): disto-dorsal claw biggest with numerous spines on distal half ventral margin, short seta, big claw with numerous spines on distal half ventral margin, longest seta, long seta and shortest disto-ventral seta.

**Maxilla** (Figs. 2M, 3J): Coxale with one plumose seta on disto-dorsal edge. Basale with two disto-ventral long plumose setae. Exopod small with three long distal plumose setae. Endopod with two articles. Article 1 long with one long and two short dorsal setae, and three disto-ventral setae. Article 2 very short, with one very small seta, two plumose setae, three claws and four plumose setae on tip. Maxilla with three endites. Endite I with seven plumose and one serrated setae. Endite II with two plumose and one serrated setae. Endite III with nine plumose and one serrated setae.

**Fifth limb** (Figs. 4A, 5A–5B): Coxale with three endites. Endite I with four plumose setae. Endite II with two plumose setae and one claw. Endite III with three plumose setae and five claws, inner claw very strong. Exopod with five articles. Article 1 with one plumose and one bare setae on mid-distal margin, main tooth comprising two slices of constituent teeth, medial teeth smooth, lateral teeth jagged. Article 2 with one long bare and one small plumose setae, and two long claws on posterior side. Article 3 with two plumose and one long bare setae on inner lobe and two short slender plumose setae on outer lobe. Articles 4 and five fused, with nine distal plumose setae. All claws of this limb with numerous disto-half ventral spines.

**Sixth limb** (Figs. 4B, 5C): Epipod with one short and three shorter long plumose setae. Endite I-IV with three, two, five and ten plumose setae, respectively. Terminal article subtriangular with about twenty-two plumose setae (some medial setae very small).

**Seventh limb**: Limb with about fifty-two articles (Figs. 4C, 5D). All articles very short. Article 40 with one disto-ventral seta with two bells. Article 41 with one disto-dorsal seta with two bells and one disto-ventral seta with three bells. Article 42 with one dorsal seta with three bells. Article 44 with one ventral seta with three bells. Article 45 with one disto-dorsal seta with three bells. Article 52 with two long setae with five and three bells respectively. Terminal article with one long and two short dorsal setae with five, four and three bells, respectively. Comb with six teeth, side opposite comb with two bare bent small pegs. Comb teeth from outside to inside from long to short sequence (Figs. 4D, 5E).

**Furca**: Furcal lamella hirsute, approximately quadrilateral with slightly inflated base, twelve claws (Figs. 4E, 5F). Claws 1, 2, 4 and 6 primary, long sickle-shaped; main claw 1 with obvious about five dorsal sawteeth, eighteen ventral sawteeth and small ventral spines (Figs. 4F, 5G); other main claws with numerous small ventral spines and bare base, from long to short in turn arrangement. Claws 3, 5, and 7–12 secondary, with several small ventral spines and bare base.

**Table 1:** Comparisons between *Euphilomedes biacutidens* sp. nov. and related species of *Euphilomedes* (♂).

Characteristics			*E. biacutidens* sp. nov.	*E. africana*Klie, 1940	*E. bradyi*Poulsen, 1962	*E. walfordi*Poulsen, 1962
Carapace	Ornamentation		Thin and transparent, tiny circle pits and setae over surface	Dense circle pits and setae over surface	Distinct small polygonal pits and setae over surface	Very hirsute, minute polygonal pits over surface
	Rostrum		Broad with rounded dorsal corner and pointed bifurcated ventral corner, incisure deep	Rounded, incisure deep	Slightly protuberant, incisure hardly indicated	Rounded, incisure shallow
	Posterior part		Postero-ventral corner protruding, posterior margin of right valve with two spines	Protruding slightly	Rounded	Rounded
	Aspect ratio		60%	68%	67%	55%
Frontal organ			Tip sharp	Base intumescent, Tip rounded	Tip thin and pointed	Tip hairy
1st antenna	Article II		Ventral and distal setae	None distal seta	Ventral, distal and medial setae	Ventral and distal setae
	Sensory seta		Thirty-two filaments	Ten filaments, and bifurcated tip	Thirty-five filaments	Less than ten filaments
	Terminal article		A-seta very short, spinose b- and g-setae very grand and long, c-seta with one mid filament, d-seta long with bifurcated tip, e- and f-setae long and bare	D- and e-setae long, f- and g-setae with several proximal and two terminal filaments	A-seta very short, b-seta with six filaments, c- and f-setae long with fifteen filaments, d- and e- setae long and bare, g-seta with four filaments	A-seta short, c- and f-setae long with thirteen filaments
2nd antenna	Endopod	III	One bent proximo-dorsal seta, twelve small ventral acute teeth, and uneven inner side	None proximo-dorsal seta, six ventral small bulbous teeth	One proximo-dorsal seta, several small ventral blunt teeth, uneven inner side	One proximo-dorsal seta, ten ventral short blunt teeth
	Exopod	I	None plumose seta	None plumose seta	One plumose seta	None plumose seta
		II	One long plumose seta	One long serrated seta	One small seta	One long serrated seta
		IX	Four long plumose setae	Four long and one short setae	Four long plumose setae	Four long and two short setae
Mandible	Coxale endite		With bifurcated tip and spines	Stout, with bifurcate tip and spines	Very reduced	Developed, two short and one long spine
	Basale		Eight ventral setae	Eleven ventral setae	Ten ventral setae	Thirteen ventral setae
	Exopod		Two equilong spinose setae	One long, one short spinose setae	One long, one very short bare setae	Two equilong spinose setae
	Endopod	I	Five ventral setae	Four ventral setae	Three ventral setae	Six ventral setae
		II	Six dorsal, five ventral setae	Nine dorsal, five ventral setae	Five dorsal, one ventral setae	Eleven dorsal, six ventral setae
		III	Two claws, four setae	Two claws, five setae	One claw, five setae	Three claws, four setae
Maxilla	Basale		Two setae	Three setae	Three setae	Two setae
	Exopod		Three long setae	Three long setae	One short and two long setae	One short and two long setae
	Endopod	I	Three distal, three disto-ventral setae	One disto-dorsal, three disto-ventral setae	Four setae	Four setae
		II	Three claws, seven setae	Two claws, nine setae	About eleven setae	Three claws, seven setae
	Endite	I	Eight setae	Six setae	Endites low bulged with few short and bare setae	Ten setae
		II	Three setae	Seven setae		Six setae
		III	Ten setae	Eight setae		Eight setae
5th limb	Exopod	II	Four setae	Four setae	Few bare setae	Five setae
		IV + V	Nine setae	Five setae	Five setae	Six setae
	Endite	I	Four setae	Six setae	Endites small with few short and bare setae	Five setae
		II	Three setae	Eight setae		Six setae
		III	Eight setae	Eleven setae		Eight setae
6th limb	Epipod		Four setae	Four setae	One seta	Three setae
	Endite	I	Three setae	Three setae	Three setae	Three setae
		II	Two setae	Four setae	Five setae	Four setae
		III	Five setae	Nine setae	Four setae	Eight setae
		IV	Ten setae	Nine setae	Five setae	Nine setae
	Terminal article		Twenty-two setae	Twenty-one setae	Ten setae	Eighteen setae
7th limb	Cleaning setae		Eleven setae	Seven setae	Nine setae	Nineteen setae
	Comb		Six teeth	Ten teeth	Five teeth	Seven teeth
	Side opposite		Two pegs	None peg	One peg	Two pegs
Furca	Claws		Twelve	Eleven	Six	Eleven
	Claw I		Obvious dorsal, ventral sawteeth and numerous ventral spines	Numerous ventral sawteeth	Numerous ventral spines	Numerous ventral sawteeth

## Discussion

According to Chen’s key of family Philomedidae Müller, 1906 (Chen & Lin, 1995), the current specimens separated from the other philomedids with the following characteristics defining the genus *Euphilomedes*: (1) the carapace is elongate oval in lateral view with pits and setae, the posterior margin is evenly rounded; (2) the rostrum is broad anteriorly, and the incisure is shallow (compared with other philomedids); (3) article 4 of the first antenna has one to four ventral setae; (4) the endopodal article 2 of the second antenna has two ventral setae; (5) the anterior triangular protuberance of the main tooth of the fifth limb has denticulate margin, the inner lobe of article 3 has three setae, and the outer lobe has two setae; (6) the seventh limb has six to nineteen cleaning setae, the comb has less than fifteen teeth; (7) the furcal lamella is not fused with the main claws, the secondary claws are alternating with the main claws, the edge between furcal lamella and claws has long cilia. With this new species, the genus *Euphilomedes* contains 32 recent species thus far (Brandão et al., 2017).

**Table 2:** Comparisons between *Euphilomedes biacutidens* sp. nov. and *Euphilomedes sinister*Kornicker, 1974.

Characteristics			*E. biacutidens* sp. nov.	*E. sinister*
Carapace	Ornamentation		Tiny circle pits	Polygonal reticulations
	Rostrum		Bifurcated ventral corner	Stout ventral corner
	Posterior part		Two spines on posterior margin of right valve	Two spines on posterior margin of left valve
	Aspect ratio		60%	66%
1st antenna	Sensory seta		About thirty-two very long filaments	Five short filaments and three long bifurcated filaments
	Terminal article		A-seta very short, spinose b- and g-setae very grand and long, c-seta with one mid filament, d-seta long with bifurcated tip, e- and f-setae long and bare	A-seta short, spinose b- and c-setae long, bare d- and e-setae long, f- and g-setae long with four short margin and to distal long filaments and bifurcated tip
2nd antenna	Exopod	II	One long plumose seta	Seta with short mid-ventral cilia
		III–V	One long plumose seta, respectively	One bare seta, respectively
		IX	Four long plumose setae	Four long, one medium, one short and one tiny setae
Mandible	Basale		Four setae in proximo-ventral group	Six setae in proximo-ventral group
	Exopod		Bare	Hirsute
	Endopod	I	Five ventral setae	Four ventral setae
		II	Six dorsal, five ventral setae	Eight dorsal, seven ventral setae
		III	Two claws, four setae	Three claws, four setae
Maxilla	Basale		Two setae	Three setae
	Exopod		Three long setae	Three long setae
	Endopod	I	Three distal, three disto-ventral setae	One disto-dorsal, five disto-ventral setae
		II	Three claws, seven setae	Twelve setae (some pectinate)
	Endite	I	Eight setae	Six setae
		II	Three setae	Eight setae
5th limb	Exopod	IV + V	Nine setae	Six setae
	Endite	I	Four setae	Six setae
		II	Three setae	Eight setae
		III	Eight setae	Eleven setae
6th limb	Epipod		Four setae	Four setae
	Endite	I	Three setae	One seta
		II	Two setae	Four setae
		III	Five setae	Eight setae
		IV	Ten setae	Eight setae
	Terminal article		Twenty-two setae	Twenty setae
7th limb	Cleaning setae		Eleven setae	Seven setae
	Comb		Six teeth	Fifteen teeth
Furca	Claws		Twelve	Thirteen
	Claw I		Five dorsal, eighteen ventral sawteeth	Numerous ventral sawteeth

Like *E. africanus* (Klie, 1940), *E. bradyi* Poulsen, 1962 and *E. walfordi* Poulsen, 1962, the new species has a row of teeth along the inner margin of article 3 of the endopod of the second antenna. However, *E. biacutidens* sp. nov. differs from these three closely related species (Table 1) in having the following combination of characteristics: (1) the carapace is thin and transparent with tiny circular pits and setae over the surface (Figs. 2A, 3A–3D); (2) the rostrum has a pointed bifurcated ventral corner (Figs. 2B, 3E); (3) the posterior margin of the right valve has one postero-dorsal spine and one postero-ventral spine (Figs. 2C, 3F); (4) the new species’ valve is more elongate than *E. walfordi* and more circular than the other two species; (5) the sensory seta of the first antenna has about thirty-two filaments (Fig. 2E); (6) there are significant differences of setae on the tip of the first antenna between these species (detailed differences are given in Table 1); (7) the endopodal article 3 of the second antenna has about twelve small ventral acute teeth and an uneven inner margin (Figs. 2F–2G); (8) the numbers of setae on the endopod of the mandible, endopod and endites of the maxilla, endopod and endites of the sixth limb have significant differences (detailed numbers are given in Table 1); (9) some setae on the fifth limb have developed into long claws (Figs. 4A, 5B); (10) the comb of the seventh limb has six teeth and the side opposite comb has two bare bend pegs (Figs. 4C–4D, 5D–5E); (11) the furcal lamella has twelve claws, the first claw has dorsal and ventral sawteeth (Figs. 4E–4F, 5F–5G).

The obvious characteristics of *E. biacutidens* sp. nov. are the postero-dorsal and postero-ventral spines on the right valve; *E. sinister* Kornicker, 1974 (including two subspecies: *E. sinister sinister* Kornicker, 1974 and *E. sinister pentathrix* Kornicker & Caraion, 1977) also shows posterior spines, which is known only in the adult female. However, both species can be easily distinguished from each other by the following remarkable differences (Table 2): (1) they have different carapace ornamentation; (2) *E. biacutidens* sp. nov. has the postero-dorsal and postero-ventral spines on the right valve (Fig. 2C, Fig. 3F), but in *E. sinister* the spines are on the left valve; (3) there are about 32 very long filaments on the sensory seta of *E. biacutidens* sp. nov., and only five short filaments and three long bifurcated filaments on the sensory seta of *E. sinister*, and there are significant differences of the setae on the tip of the first antenna between these species (detailed differences are given in Table 2); (4) *E. biacutidens* sp. nov. has two mandibular claws, *E. sinister* has three; (5) they have significant differences in the numbers of setae on the endites of the maxillae and the fifth limbs (except endite III of maxilla, with detailed numbers given in Table 2); (6) *E. biacutidens* sp. nov.has more cleaning setae on the seventh limb, but fewer teeth on the comb (Figs. 4C–4D, 5D–5E).

Additionally, *E. biacutidens* sp. nov. shows some long claws instead of setae on the fifth limb (Figs. 4A, 5B); this is a diagnostic characteristic of the species and is an unusual characteristic in the genus. The rostrum has a pointed bifurcated ventral corner (Figs. 2B, 3E), which is also a distinctive characteristic not previously observed in the genus.

Finally, the distance between the sampling localities of the holotype and paratypes indicates that the new species may be widely distributed southeast off China (Fig. 1).

## Additional Information and Declarations

### New Species Registration

The following information was supplied regarding the registration of a newly described species: Publication LSID: urn:lsid:zoobank.org:pub:557FB253-93C6-473E-9E07-227D9D9C1A60 *Euphilomedes biacutidens* LSID: urn:lsid:zoobank.org:act:F773B126-7B58-45EA-8F28-8FCEB23B5868.
